# Privacy-Enhancing *k*-Nearest Neighbors Search over Mobile Social Networks †

**DOI:** 10.3390/s21123994

**Published:** 2021-06-09

**Authors:** Yuxi Li, Fucai Zhou, Yue Ge, Zifeng Xu

**Affiliations:** 1 School of Computer Science and Engineering, Northeastern University, Shenyang 110819, China; 2 Software College, Northeastern University, Shenyang 110819, China; fczhou@mail.neu.edu.cn (F.Z.); yggeyue@foxmail.com (Y.G.); 3 School of Cybergram, Hainan University, Haikou 570228, China; tnimdk@gmail.com

**Keywords:** mobile social networks, privacy-enhancing, collaboration architecture, location search, secure multi-party computation, homomorphic encryption

## Abstract

Focusing on the diversified demands of location privacy in mobile social networks (MSNs), we propose a privacy-enhancing *k*-nearest neighbors search scheme over MSNs. First, we construct a dual-server architecture that incorporates location privacy and fine-grained access control. Under the above architecture, we design a lightweight location encryption algorithm to achieve a minimal cost to the user. We also propose a location re-encryption protocol and an encrypted location search protocol based on secure multi-party computation and homomorphic encryption mechanism, which achieve accurate and secure *k*-nearest friends retrieval. Moreover, to satisfy fine-grained access control requirements, we propose a dynamic friends management mechanism based on public-key broadcast encryption. It enables users to grant/revoke others’ search right without updating their friends’ keys, realizing constant-time authentication. Security analysis shows that the proposed scheme satisfies adaptive L-semantic security and revocation security under a random oracle model. In terms of performance, compared with the related works with single server architecture, the proposed scheme reduces the leakage of the location information, search pattern and the user–server communication cost. Our results show that a decentralized and end-to-end encrypted *k*-nearest neighbors search over MSNs is not only possible in theory, but also feasible in real-world MSNs collaboration deployment with resource-constrained mobile devices and highly iterative location update demands.

## 1. Motivation

With the rapid development of 5G Wireless Communication, mobile social networks (MSNs), represented by instant messaging and location sharing, have become essential parts of people’s everyday lives. According to [1], the number of enrolled users in MSNs worldwide reaches 862 million in 2020, and it is estimated to exceed 900 million by the end of 2021. In particular, the utilization rate of location-based MSN services reaches 96.9% based on the positioning system (e.g., GPS, WiFi, Bluetooth, etc.) embedded in mobile devices, such as Facebook’s “Nearby friends”, Foursquare’s “Swarm”, and Joyrun’s “real-time running competition”, and so forth. In these services, users can broadcast their locations among friends and send location-based queries for nearby friends. Therefore, the location-based services provide a profoundly mobile interface for users’ real-life social networks.

Nevertheless, people are using the enormously popular MSNs services without realizing their privacy concerns: the MSNs services providers can observe and accumulate the geo-location that users transmit through the network. According to the Mobile APP Security Research [2], among the 50 MSN services surveyed, there are 35 services that leak users’ location data to advertisers or data analysis services on purpose without any permission. In recent years, a lot of research also uses data analysis and machine learning technology to extract a large number of their sensitive information from users’ location information over MSNs: by analyzing their search patterns, the matched friends and search similarities and so forth, it is easy to predict the location conversion patterns between users and their friends [3,4]. It is also turned out that Facebook’s historical spatiotemporal trajectory leaks the geographical distance between each user and the spot that he frequently queries, and then the service provider can learn the access probability—whether and when the user will check-in the next time [5].

To avoid illegal access to users’ locations and search patterns by unauthorized service providers and hackers, previous research aimed to encrypt the location information before uploading. However, traditional encryption methods limit the MSNs service provider’s ability to provide location-based services for users. To achieve privacy-preserving location-based query, the straightforward mathematical methods deploy private information retrieval [6], searchable encryption [7] and other crypto primitives to make the encrypted location data searchable. However, these methods come at the huge cost of computation and communication overhead. Moreover, in a privacy-preserving setting, users have their keys embedded in their mobile devices. If a user is allowed to share his/her location encryption key with friends, he/she needs to launch a search request to the platform multiple times when he wants to retrieve his/her friends’ locations. Moreover, when granting or revoking friends’ search rights, a user and his/her friends should update their keys with synchronous locally, which are not suitable for MSNs platforms with highly extensible requirements. To the best of our knowledge, it is fair to say that achieving fine-grained location access control while providing an efficient, secure location-based neighbors search service has become one of the challenging research topics in the field of privacy-enhancing MSNs and still remains open.

In this work, we translate the high-level vision of the above issues and location privacy demand in MSNs into technical requirements and design a privacy-enhancing *k*-nearest neighbors search scheme containing cryptographic protocols that meet them. The purpose of this work is to protect users’ location data and search patterns privacy, and make it available for users to query for their *k*-nearest neighbors based on current distances. In terms of technical contribution, our work presents an efficient construction so that the server can effectively compute and sort the encrypted distance between a user and his/her friends without any decryption operation, which is the first to tackle this problem through the lens of secure multi-party computation. It also achieves lightweight friend authentication and authority management by enabling users to grant/revoke their friends’ search rights without updating others’ keys. In terms of security, our scheme satisfies adaptive L-semantic secure and revocation secure under random oracle model. We also undertook an extensive experiment that validates our work, showing that the proposed scheme is possible in theory and feasible in practice.

## 2. Related Works

### 2.1. MSNs Privacy

In recent decades, researchers have proposed many privacy-preserving approaches for MSNs. Encryption is the most common method for achieving privacy. For example, Flybynight [8] is a Facebook application for encrypting and decrypting sensitive data using client-side JavaScript. However, it is easy to be attacked by an adversary because the server holds users’ keys and takes charge of key management. NOYB (short for none of your business) [9] offers privacy and preserves MSN services’ functionality based on a secret dictionary’s encryption. Besides, there have been many privacy-preserving matching solutions over MSNs proposed with different techniques. Some schemes are based on private set intersection protocols [10,11] to allow two users to compute the intersection of the two private profile sets privately, but leak no useful information of both parties. For example, Niu et al. designed a spatiotemporal matching scheme for privacy-aware users in MSNs based on the profile’s weight or level and the participant’s social strength [10]. Zhang et al. proposed the concept of a fine-grained privacy information matching protocol by giving preference to each profile and using a similarity function to measure the matching degree [11]. To reduce computation cost, some works [12,13] designed non-encryption-based privacy-preserving matching protocols. Fu et al. proposed a privacy-preserving common-friend matching scheme based on a bloom filter [12]. It transmitted the common profiles of two users into an intersection of bloom filters, which ensures the privacy of friend lists against unknown users. However, it will not be able to resist brute-force attacks, resulting in privacy information leakage. Sun et al. [13] proposed a privacy-preserving spatiotemporal profiles matching scheme to let each user periodically record his locations by a geographic cell index among a large set of predefined ones, which can ensure spatiotemporal privacy at the cost of possibly huge communication and computation overhead.

### 2.2. Location Privacy

With the rapid development and enormous popularity of location-based services, scholars have paid more and more attention to location data’s privacy and security. Many approaches focus on how to perform privacy-preserving location queries: Bamba et al. proposed a *k*-anonymity-based scheme that relies on a server to construct an anonymous set based on users’ original queries to make query indistinguishable on the server-side [14]. Bordenabe et al. [15] and Shi et al. [16] both integrated differential privacy to realize nearby friends’ queries. Differential privacy provides a rigorous privacy guarantee by adding noise (randomly to choose a set of fake locations ) to make their data and query deferentially private. Jorgensen et al. incorporated a clustering procedure that groups users according to the social network’s natural community structure and significantly reduced noise [17]. The above works [14,15,16,17] can achieve relatively high efficiency. However, the limitations of these works are that it is challenging to achieve provable security guarantees with formal security definitions, since they did not employ well-designed and provable encryption methods. Zhou et al. took advantage of private information retrieval (PIR) to realize nearby friends’ queries [18]. It provides strong cryptographic guarantees but needs complex operations, and it only protects query privacy but not location privacy. Li et al. designed a private location information matching protocol over MSNs based on inner product similarity (IPS) [19], putting users’ map locations into vectors and encrypting the vectors. The similarity function is used to measure the similarity degree of the encrypted vectors of different users. Schlegel et al. designed an encryption method of dynamic location grid index structure [20], achieving neighbor point search on the premise of not revealing location privacy to the third party. In the above encryption-based schemes, the computation efficiencies are not ideal, requiring multi-round interactions at the logarithmic level between user and server.

Many other works are based on higher security assumptions to achieve a trade-off between security and efficiency. For example, some works [21,22] assumed that the service provider is honest and that it has the authority to access the location plaintext without leaking any information to others. Some works [21,23,24] introduced a trusted third party (TTP) to achieve the trade-off between security and efficiency. Unfortunately, there may not exist such a TTP in real MSNs scenarios. Some non-TTP solutions [15,20] are based on approximate measurements (e.g., linear programming and dynamic grid) with no accurate result. Some works [18,25] need complex operations (e.g., sending fake queries or receiving redundant results) to achieve secure guarantees, which incur high communication and computation overhead at the user-side, making them unsuitable for resource-constrained mobile devices.

## 3. The Proposed Scheme

### 3.1. Overview

The privacy-enhancing *k*-nearest neighbors search scheme over MSNs can be viewed as a decentralized system of end-to-end encrypted social network databases, focusing on the diversified demands of location privacy in MSNs. Our design relies on various cryptographic building blocks, including pseudo-random function, homomorphic crypto mechanism, secure multi-party computation and broadcast encryption.

- Aiming at the limited computation power of resource-constrained mobile devices, we design a lightweight end-to-end location encryption algorithm and a server-aid location re-encryption protocol based on Paillier homomorphic encryption to achieve further location sharing. The protocol allows the service provider to transfer friends’ location ciphertexts into the query user’s homomorphic ciphertexts without requiring them to be online to participate in the calculation.
- We build a secure dual-server architecture and design a secure *k*-nearest neighbors search protocol by secure multi-party computation and a homomorphic encryption mechanism under this architecture. The server can effectively compute and sort the distance between users and their friends without any decryption operation. Compared with the cloud-center model, where a single server holds complete knowledge, the dual-server architecture minimizes the leakage to the servers and reduces the cost of communication between the mobile user and the server.
- To achieve fine-grained access control, we design a dynamic friends management mechanism based on public-key broadcast encryption. It enables users to grant/revoke their friends’ search rights without updating others’ keys, achieving lightweight friend authentication and authority management. Moreover, this mechanism satisfies revocation secure that the adversary cannot obtain the user’s location information through collusion with the server and the revoked friends, thus further improving the scheme’s overall security.

### 3.2. Architecture and Syntax

Our scheme is designed to be executed among: **U**, $S_{1}$, and $S_{2}$. **U** is a set that contains *n* mobile users $\left\{U_{1},\ldots ,U_{n}\right\}$. Each user $U_{i}\in U$ can connect with others as his friends dynamically. $S_{1}$ is the primary server that provides a mobile social network service to all users in **U**. Each user $U_{i}\in U$ can send a search request to $S_{1}$ for *k*-nearest neighbors among friends based on current location. $S_{2}$ is a collaborated server to conduct secure computation with $S_{1}$ for *k*-nearest neighbors search.

**Figure 1:**
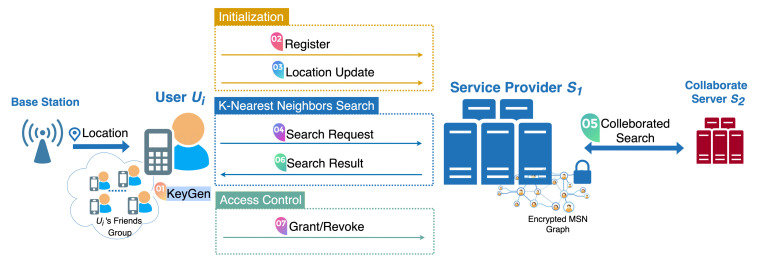
The architecture.

The scheme’s architecture is shown in Figure 1. At a high level, users’ information and their relationships are modeled by a direct graph structure G. To initialize the system, the primary server $S_{1}$ executes **Initial** algorithm to output public parameter params and an empty G. Any user $U_{i}$ should use public parameter params to generate his symmetric key $K_{i}$ and public/secret keys $\left(PK_{i},SK_{i}\right)$ locally by executing KeyGen algorithm and interacts with the primary server $S_{1}$ for registration by **Join** protocol. Any enrolled user $U_{i}\in U$ can grant/revoke $U_{j}\in U$’s location search right by interacting with $S_{1}$ in **Grant/Revoke** protocols. $U_{i}$ holds a friends index $F_{i}$ that records his granted friends. According to the real-life MSNs’ location service architecture, we deploy trusted location infrastructure to provide tracing service by sending the current location $l_{i}$ of each user $U_{i}\in U$ to his local mobile device periodically. $U_{i}$ executes LocUpdate to encrypt his location data $l_{i}$ by his symmetric key $K_{i}$ at local and uploads the location ciphertext $C_{i}$ to $S_{1}$. $U_{i}$ then can execute **Search** protocol with $S_{1}$ by sending *k*-nearest neighbors search request. $S_{1}$ performs encrypted search in G with the assistance of $S_{2}$ and returns the search result to $U_{i}$, without relying on the presence of any other user. The proposed scheme’s syntax consists of seven polynomial-time algorithms and protocols, which is shown in **Syntax** below:


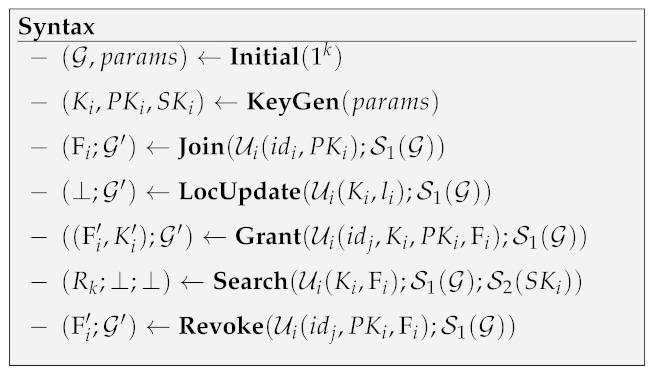

**Definition** **1**  (Correctness). *Correctness implies that, for all $1^{k}$, all (G,params) generated by **Initial**$\left(1^{k}\right)$, all $\left(K_{i},PK_{i},SK_{i}\right)$ generated by **KeyGen**(params), all $\left(F_{i};G^{\prime }\right)$ generated by $\mathrm{Join}(U_{i}\left(id_{i},PK_{i}\right);S_{1}\left(G\right))$, all $\left(K_{i},PK_{i},SK_{i}\right)$ generated by**KeyGen**(params), and all sequences of **LocUpdate**, **Grant** and **Revoke** protocols, $\mathrm{Search}(U_{i}\left(K_{i},F_{i}\right);S_{1}\left(G\right);S_{2}\left(SK_{i}\right))$ will always output result $R_{k}$ that: $R_{k}$ satisfies $D_{1}<\ldots <D_{K}$; and there does not exist $U_{i}\in R_{k}$ such that $U_{i}\notin F_{s}$ and $U_{i}\in \left\{F_{s}\setminus R_{k}\right\}$ that $D_{i}<max_{U_{j}\in R_{k}}\left\{D_{j}\right\}$.*

### 3.3. Security Definition

#### 3.3.1. Adaptive L-Semantic Secure

The security definition of adaptive L-semantic secure is formalized by an ideal/real-world paradigm [7]. Roughly speaking, we require that the execution of the scheme in the real-world is indistinguishable from an ideal-world. In real-world Real$\left(1^{k}\right)$, the protocols between the adversarial servers and the user execute just like in the real scheme. In ideal-world Ideal$\left(1^{k}\right)$, there exist two simulators $Sim_{1}$ and $Sim_{2}$ that can obtain the leakage information from leakage functions and try to simulate the execution of $A_{1}$ and $A_{2}$ in Real$\left(1^{k}\right)$.

**Definition** **2**  (Adaptive L-Semantic Secure). *Given the syntax in Section 3.2 and considering the following probabilistic paradigms, where **U**= $\left\{U_{1},\ldots ,U_{n}\right\}$ is the users’ set, $A_{1}$ and $A_{2}$ are two non-colluding adversaries with pseudo-random polynomial time (PPT) computation ability, $Sim_{1}$ and $Sim_{2}$ are two PPT simulators and $L_{1}$ to $L_{4}$ are leakage functions.*

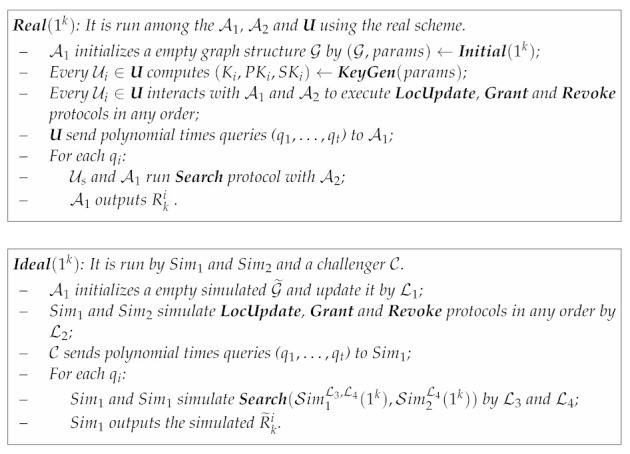

*The proposed scheme achieves adaptive L-semantic security if, for all polynomial time $A_{1}$ and $A_{2}$, there exists polynomial time simulators $Sim_{1}$ and $Sim_{2}$ such that the following two distribution ensembles are computationally indistinguishable:*

$${\mathrm{Output}}_{A_{1/2}}^{\mathrm{Real}(1^{k})}\approx {\mathrm{Output}}_{Sim_{1/2}}^{\mathrm{Ideal}(1^{k})}.$$

#### 3.3.2. Revocation Security

Revocation security guarantees that the scheme satisfies that any user’s revoked friend cannot provide a valid search for his location, even if an adversary illegally steals the revoked friend’s key. We construct the experiment ${\mathrm{Exp}}_{A_{rev}}^{\mathrm{Revoke}}\left(1^{k}\right)$ to formalize the revocation security definition. ${\mathrm{Exp}}_{A_{rev}}^{\mathrm{Revoke}}\left(1^{k}\right)$ is interactively executed by a challenger C and an adversary $A_{rev}$ who has the ability to add friends, perform a search and revoke friends in the real scheme. C deletes the user who has been added to the friends index by $A_{rev}$. $A_{rev}$ continues to generate a search token using the revoked friend’s identity and makes a search request. After a polynomial number of queries, C revokes all users that are queried to the Grant oracle but are not subsequently queried to the Revoke oracle (i.e., all users for which $A_{rev}$ holds their valid user keys).

The adversary $A_{rev}$ must then produce a search token which, when used as an input to **Search** protocol, does not produce null, that is, $A_{rev}$ must produce a valid search request even though it does not hold a non-revoked key. After several rounds of queries, if $A_{rev}$’s probability of winning the revocation security experiment with PPT computation ability is negligible, then we can say that the proposed scheme satisfies revocation security.

**Definition** **3**  (Revocation Secure). *Given the syntax in Section 3.2 and considering ${\mathrm{Exp}}_{A_{rev}}^{\mathrm{Revoke}}\left(1^{k}\right)$, which is executed by a challenger C and an adversary $A_{rev}$:*

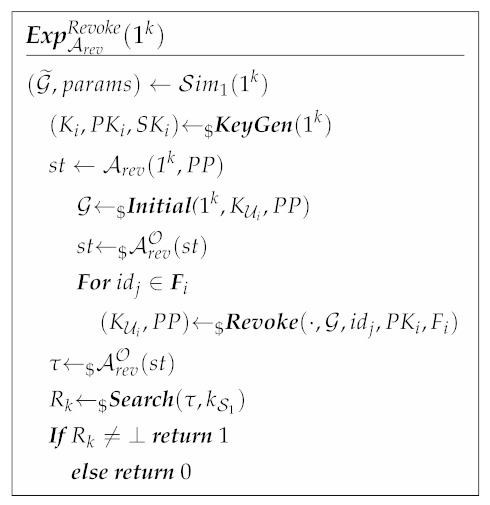

*Specifically, C runs Initial to initialize G, generates key $\left(K_{i},PK_{i},SK_{i}\right)$ and state ciphertext $cst_{i}$ by KeyGen and Join. C sends G and $cst_{i}$ to $A_{rev}$. $A_{rev}$ can access to the following oracles, where · denotes the parameters that are provided by $A_{rev}$ himself:*

- *$O_{\mathrm{Grant}}\left(\;\cdot \;,G,id_{j},PK_{i},F_{i}\right)$: $A_{rev}$ can send grant friend request to this oracle. If $id_{j}\notin F_{i}$, then the oracle $O_{\mathrm{Grant}}$ runs Grant by the input provides by $A_{rev}$. If $id_{j}\in F_{i}$, then the oracle $O_{\mathrm{Revoke}}$ outputs *⊥*.*
- *$O_{\mathrm{Revoke}}\left(\;\cdot \;,G,id_{j},PK_{i},F_{i}\right)$: $A_{rev}$ can send revoke friend request to this oracle. If $id_{j}\in F_{i}$, then the oracle $O_{\mathrm{Revoke}}$ runs Revoke by the input provides by $A_{rev}$. If $id_{j}\notin F_{i}$, then the oracle $O_{\mathrm{Revoke}}$ outputs *⊥*.*
- *$O_{\mathrm{Search}}\left(\;\cdot \;,G,PK_{s},F_{s}\right)$: $A_{rev}$ can send a search request in G to this oracle. $A_{rev}$ generates a search token and sends it to $O_{\mathrm{Search}}$. Then, the oracle $O_{\mathrm{Search}}$ runs Search by the input provides by $A_{rev}$, and outputs the search result to $A_{rev}$.*

*After polynomial times rounds of queries, C revokes all the users that have access to $O_{\mathrm{Grant}}\left(\;\cdot \;,G,id_{j},PK_{i},F_{i}\right)$ but not $O_{\mathrm{Revoke}}\left(\;\cdot \;,G,id_{j},PK_{i},F_{i}\right)$. $A_{rev}$ generates a search token τ in Search protocol. If the output of Search is not *⊥*, then returns 1, otherwise returns 0.*

*The proposed scheme achieves revocation security if, for all $A_{rev}$, all $1^{k}$, the advantage of $A_{rev}$ to win ${\mathrm{Exp}}_{A}^{\mathrm{Revoke}}\left(1^{k}\right)$ is negligible:*

$$|\mathrm{Pr}\left[{\mathrm{Exp}}_{A_{rev}}^{\mathrm{Revoke}}\left(1^{k}\right)=1\right]|\leq \mathrm{negl}\left(1^{k}\right).$$

### 3.4. The Detailed Construction

Let BE={BE.KeyGen,BE.Join,BE.Enc,BE.Dec} be a broadcast encryption scheme that retains CPA secure against a coalition of revoked users [26], P={P.KeyGen,P.

Enc,P.Dec} be the Pallier encryption scheme [27], GM={GM.KeyGen,GM.Enc,GM.Dec} be the Goldwasser-Micali encryption scheme [28], and $F:{\left\{0,1\right\}}^{k}\times {\left\{0,1\right\}}^{*}\to {\left\{0,1\right\}}^{k}$ be a pseudo-random function. The detailed construction is given as follows:

#### 3.4.1. Initialization

On input of the security parameter $1^{k}$, $S_{1}$ initializes the global social network graph structure G=(V,E) and public parameters params. In graph G, the maximal number of vertexes in V is *n*, that is |V|=n, which represents the maximum amount of enrolled users. Each vertex $v_{i}\in V$ should be attached with the information for an enrolled user $U_{i}\in U$ that $S_{1}$ gathered. The existence of a non-zero edge $e_{ij}\in E$ between $v_{i}\in V$ and $v_{j}\in V$ represents the friends relationship of $U_{i}$ and $U_{j}$. In other words, if $U_{i}$ and $U_{j}$ are strangers to each other, then $e_{ij}=0$. G is empty at initialization.

#### 3.4.2. Key Generation

If a user $U_{i}$ is willing to join in the system, he should generate his own keys at local. $U_{i}$’s keys consists of the following parts: the key for the pseudo-random function F to encrypt location data, the key pair for the broadcast encryption scheme BE, the key pairs for the Pallier encryption scheme P and the Goldwasser-Micali encryption scheme GM. $U_{i}$ first takes as input the binary representation of the public parameters params, and randomly selects a *k*-bit string $k_{i}\in {\left\{0,1\right\}}^{k}$ for his key of F. Then he generates $\left(bpk_{i},msk_{i}\right)$ by BE.KeyGen, $\left(pk_{i},sk_{i}\right)$ by P.KeyGen and $\left(pk_{i}^{\prime },sk_{i}^{\prime }\right)$ by GM.KeyGen. Afterwards, he forms his symmetric key $K_{i}$ as $\left(msk_{i},k_{i}\right)$, public key $PK_{i}$ as $\left(bpk_{i},pk_{i},pk_{i}^{\prime }\right)$ and secret key $SK_{i}$ as $\left(sk_{i},sk_{i}^{\prime }\right)$. The lengths of the above keys are determined by the security parameter $1^{k}$. Finally, $U_{i}$ publishes his public key $PK_{i}$ throughout the system.

#### 3.4.3. Join

Before joining in, $U_{i}$ should generate his friends index $F_{i}$ with *d* entries, where *d* represents the maximum amount of $U_{i}$’s friends. $F_{i}$ is a key-value data structure, which is empty at first. The key part of $F_{i}$ will be attached with the granted friends’ identities, the corresponding value part will be attached with the granted friends’ session keys. More precisely, if $U_{j}$ is a friend of $U_{i}$, then $F_{i}\left[id_{j}\right]$ stores the session key $k_{j}^{i}$ that $U_{j}$ has shared with $U_{i}$, where $id_{i}$ represents $U_{i}$’s identity: $F_{i}\left[id_{j}\right]=k_{j}^{i}$, where $id_{j}$ represents $U_{j}$’s identity. To register, $U_{i}$ should also add the server $S_{1}$ in $F_{i}$ by generating $S_{1}$’s session key $k_{S_{1}}^{i}$ by $\mathrm{BE}.Join_{msk_{i}}\left(S_{1}\right)$ and setting $F_{i}\left[S_{1}\right]=k_{S_{1}}^{i}$. Afterwards, $U_{i}$ randomly selects a *k*-bit string $st_{i}$ as his current state value and encrypts $st_{i}$ to $cst_{i}$ by $\mathrm{BE}.Enc_{bpk_{i}}\left(S_{1},st_{i}\right)$. Then $U_{i}$ sends $S_{1}$ a registration request $Re_{i}=(id_{i}||cst_{i}\left|\right|k_{S_{1}}^{i})$. $S_{1}$ selects an empty vertex $v_{i}\in V$ in G and attaches $v_{i}$ with $Re_{i}$.

#### 3.4.4. Location Update

An enrolled user $U_{i}\in U$ can interact with $S_{1}$ to update his location by **LocUpdate** protocol. First, $U_{i}$ obtains his current geo-location $l_{i}$ from the trusted location infrastructure that sends $U_{i}$’s geo-location to his local mobile device periodically. $U_{i}$ maps $l_{i}$ into an integer $x_{i}$ from $Z_{k}$ and computes its square ${x_{i}}^{2}$. To hide $l_{i}$ from $S_{1}$, $U_{i}$ needs to encrypt $x_{i}$ and ${x_{i}}^{2}$ at local: he chooses two random values $r_{1}$ and $r_{2}$ from $Z_{k}$, uses his key $k_{i}$ to generate $p_{1}=F_{k_{i}}\left(r_{1}\right)$ and $p_{2}=F_{i}\left(r_{2}\right)$ by pseudo-random function F, and hides $x_{i}$ and ${x_{i}}^{2}$ into $c_{x_{i}}=\left(x_{i}+p_{1},r_{1}\right)$ and $c_{{x_{i}}^{2}}=\left({x_{i}}^{2}+p_{2},r_{2}\right)$ by $\left(p_{1},p_{2}\right)$ and $\left(r_{1},r_{2}\right)$. Finally he forms his current location ciphertext $L_{i}$ as $L_{i}=\left(c_{x_{i}},c_{x_{i}^{2}}\right)$ and sends $L_{i}$ to $S_{1}$. $S_{1}$ updates the information embedded in vertex $v_{i}$ in G as $v_{i}\leftarrow v_{i}\left|\right|\left\{L_{i}\right\}$.

#### 3.4.5. Grant

When $U_{i}$ connects $U_{j}$ as his friend, he should grant $U_{j}$’s right to search his location by conducting **Grant** protocol with $S_{1}$. First, $U_{i}$ adds $U_{j}$’s identity $id_{j}$ as an entry in $U_{i}$’s friends index $F_{i}$, generates $U_{j}$’s session key $k_{j}^{i}$ by $\mathrm{BE}.Join_{msk_{i}}\left(id_{j}\right)$, sends $k_{j}^{i}$ to $U_{j}$ in secure channel. $U_{i}$ then selects a *k*-bit string $st_{i}^{\prime }$ as his updated state value, encrypts $st_{i}^{\prime }$ to $cst_{i}^{\prime }$ for the updated friends group in $F_{i}$ that contains $U_{j}$ by $\mathrm{BE}.Enc_{\left(bpk_{i}\right)}\left(st_{i}^{\prime },F_{i}\right)$, and boardcasts $cst_{i}^{\prime }$ to the system. After receiving his session key $k_{i}^{j}$ from $U_{j}$, he attaches $F_{i}\left[id_{j}\right]$ with $k_{i}^{j}$: $F_{i}\left[id_{j}\right]=k_{i}^{j}$. Afterwards, he sends grant request $\left(cst_{i}^{\prime }||id_{j}\right)$ to $S_{1}$. $S_{1}$ first checks whether there is a non-zero direct edge $e_{ij}$ in G. If not, it sets $e_{ij}=1$ and update $v_{i}$ in G with new $cst_{i}^{\prime }$: $v_{i}\leftarrow v_{i}\setminus \left\{cst_{i}\right\}\cup \left\{cst_{i}^{\prime }\right\}$.

#### 3.4.6. K-Nearest Neighbors Search

Each enrolled user $U_{s}\in U$ can send a search request to $S_{1}$ for retrieving his *k*-nearest neighbors sorted by distances, shown in Protocol 1. First of all, $U_{s}$ retrieves his friends’ identities $\left\{id_{1},\ldots ,id_{d}\right\}$ from his friends index $F_{i}$, downloads the state ciphertexts $\left\{cst_{1},\ldots ,cst_{d}\right\}$ for all his friends $\left\{U_{1},\ldots ,U_{d}\right\}$ from the system. For each $cst_{i}\in \left\{cst_{1},\ldots ,cst_{d}\right\}$, $U_{s}$ decrypts it to $st_{i}^{\prime }$ by $\mathrm{BE}.Dec_{k_{s}^{i}}\left(st_{i}^{\prime }\right)$. Afterwards, $U_{s}$ consolidates the decryption results into search token $\tau =(st_{1}^{\prime },\ldots ,st_{d}^{\prime })$ and sends τ to $S_{1}$. After receiving τ, $S_{1}$ extracts $\left\{cst_{1},\ldots ,cst_{d}\right\}$ from $v_{j}$’s all adjacents $\left\{v_{1},\ldots ,v_{d}\right\}$ in G. For each $cst_{i}\in \left\{cst_{1},\ldots ,cst_{d}\right\}$, $S_{1}$ decrypts it to $st_{i}$ by $\mathrm{BE}.Dec_{k_{S_{1}}^{i}}\left(cst_{i}\right)$. It compares each $st_{i}$ in $\left\{st_{1},\ldots ,st_{d}\right\}$ with $st_{i}^{\prime }$ in τ: if $st_{j}^{\prime }$ is equal to $st_{i}$, then $U_{s}$ has been granted the right to search for $U_{i}$’s location. Afterwards, for each granted $U_{i}$, $S_{1}$ retrieves the encrypted location $L_{i}$ attached in $v_{i}$. Then, $S_{1}$ and $S_{2}$ conduct the following protocols:


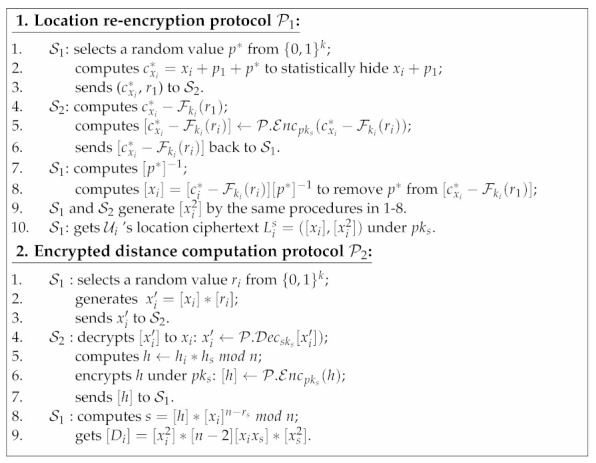


After conducting $P_{1}$ and $P_{2}$ for all $U_{s}$’s friends, $S_{1}$ forms a key-value set $I=\{\left(id_{1},\left[D_{1}\right]\right),\ldots ,\left(id_{d},\left[D_{d}\right]\right)\}$ that contains all pairs of the encrypted distances between $U_{s}$ and his friends along with their identities. $S_{1}$ encrypts each $id_{i}\in I$ to $\left[id_{i}\right]$ by $P.Enc_{pk_{s}}\left(id_{i}\right)$, and generates $\tilde{R}=\left\{\left(\left[id_{1}\right],\left[D_{1}\right]\right),\ldots ,\left(\left[id_{d}\right],\left[D_{d}\right]\right)\right\}$. $S_{1}$ and $S_{2}$ perform a secure comparison protocol $P_{3}$ [29] for $S_{1}$ and $S_{2}$ to compare each pair $\left(\left[id_{x}\right],\left[D_{x}\right]\right)$ and $\left(\left[id_{y}\right],\left[D_{y}\right]\right)$ in $\tilde{R}$ based on the distance $D_{x}$ and $D_{y}$. We use $P_{3}$ as a black-box building block for **Search** protocol, and pick Batcher’s sorting [30] for performing efficient parallel multi-time comparisons.

Finally, $S_{1}$ obtains the sorted final result $R=\{\left(\left[id_{1}\right],\left[D_{1}\right]\right),\ldots ,\left(\left[id_{d}\right],\left[D_{d}\right]\right)\}$, and sends it back to $U_{s}$. $U_{s}$ can decrypt each $\left[id_{i}\right]$ to $id_{i}$ by $P.Dec_{sk_{s}}\left[id_{i}\right]$, then obtain his *k*-nearest neighbors identities $R_{k}=\left(id_{1},\ldots ,id_{k}\right)$ that were sorted by distance.

#### 3.4.7. Revoke

When $U_{i}$ wants to revoke $U_{j}$’s search right, he should conduct **Revoke** protocol with $S_{1}$. $U_{i}$ first deletes $F_{i}\left[id_{j}\right]$ locally, selects a *k*-bit string $st_{i}^{\prime }$ as his updated state value, encrypts $st_{i}^{\prime }$ to $cst_{i}^{\prime }$ by $\mathrm{BE}.Enc_{bpk_{i}}\left(st_{i}^{\prime },F_{i}\right)$ for the updated group in $F_{i}$ that excludes $U_{j}$. Afterwards, he sends revoke request $\left(cst_{i}^{\prime }||id_{j}\right)$ to $S_{1}$. $S_{1}$ first checks whether there is a non-zero direct edge $e_{ij}$ in G. If true, it set $e_{ij}=0$ and update $v_{i}$ in G with new $cst_{i}^{\prime }$: $v_{i}\leftarrow v_{i}\setminus \left\{cst_{i}\right\}\cup \left\{cst_{i}^{\prime }\right\}$.


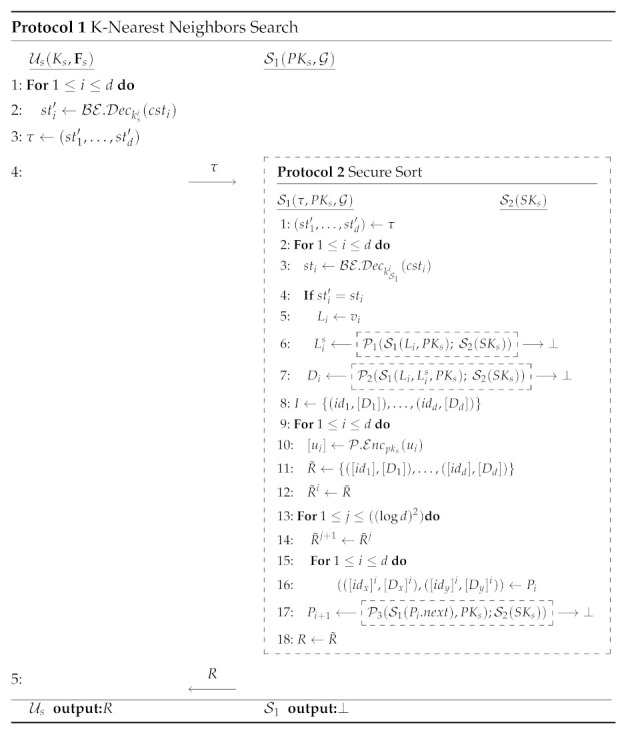


## 4. Security Analysis

### 4.1. Adaptive L-Semantic Secure

**Theorem** **1.**  *If F is a pseudo-random function, P, GM and BE are CPA secure, and the DGK protocol [31] is proved to be semantically secure in the random oracle model, then the proposed scheme satisfies adaptive L-semantic security, which is defined in Definition 2.*

**Proof.**  We construct two simulators $Sim_{1},Sim_{2}$ that can generate the simulated values in $\mathrm{Ideal}(1^{k})$ using the information given in the leakage functions $L_{1}$ to $L_{4}$, and prove that $\mathrm{Ideal}(1^{k})$ is indistinguishable with Real$\left(1^{k}\right)$ by any PPT adversary.

Given the information leaked from $L_{1}$, $Sim_{1}$ can learn $|cst_{i}^{j}|$ and $\left\{|cst_{i}^{1}|,\ldots ,|cst_{i}^{q}|\right\}$. Afterwords, it can choose random value ${\tilde{cst}}_{i}^{j}$ with lengths $|cst_{i}^{j}|$ to simulate $cst_{i}^{j}$. Due to the CPA secure of BE, ${cst}_{i}^{j}$ is indistinguishable from ${\tilde{cst}}_{i}^{j}$ by any PPT adversary. Therefore, $Sim_{1}$ cannot learn extra information from $\left\{|cst_{i}^{1}|,\ldots ,|cst_{i}^{q}|\right\}$, which satisfies:

$${\mathrm{Output}}_{A_{1}}^{\mathrm{Real}}\left(\left\{cst_{i}^{1},\ldots ,cst_{i}^{q}\right\}\right)\approx {\mathrm{Output}}_{Sim_{1}}^{\mathrm{Ideal}}\left(\left\{{\tilde{cst}}_{i}^{1},\ldots ,{\tilde{cst}}_{i}^{q}\right\}\right).$$

Given the information leaked from $L_{2}$, $Sim_{1}$ can learn $|c_{x_{i}}^{j}|$ and $|c_{{x_{i}}^{2}}^{j}|$ in $L_{i}^{j}=\left(c_{x_{i}}^{j},c_{{x_{i}}^{2}}^{j}\right)$. Afterwards, it can choose two random values in lengths $|c_{x_{i}}^{j}|$ and $|c_{{x_{i}}^{2}}^{j}|$ to output the simulated ${\tilde{L}}_{i}^{j}=\left({\tilde{c}}_{x_{i}}^{j},{\tilde{c}}_{{x_{i}}^{2}}^{j}\right)$. Since $L_{i}^{j}$ is generated by F, ${\tilde{L}}_{i}^{j}$ and $L_{i}^{j}$ are indistinguishable by any PPT adversary due to the randomness of F. Therefore, $Sim_{1}$ cannot learn extra information from the update history $\left\{L_{i}^{1},\ldots ,L_{i}^{q}\right\}$, which satisfies:

$${\mathrm{Output}}_{A_{1}}^{\mathrm{Real}}\left(\left\{L_{i}^{1},\ldots ,L_{i}^{q}\right\}\right)\approx {\mathrm{Output}}_{Sim_{1}}^{\mathrm{Ideal}}\left(\left\{{\tilde{L}}_{i}^{1},\ldots ,{\tilde{L}}_{i}^{q}\right\}\right).$$

Given the information leaked from $L_{3}$, $Sim_{1}$ can obtain search tokens $\left\{\tau_{1},\ldots ,\tau_{q}\right\}$. Afterwards, it can choose random value ${\tilde{\tau }}_{i}$ in size $|\tau_{i}|$ to simulate each $\tau_{i}$. Moreover, since $\left\{st_{1}^{\prime },\ldots ,st_{d}^{\prime }\right)$ is generated by BE.Dec by decrypting $\left\{cst_{1},\ldots ,cst_{d}\right\}$ using keys $\left\{k_{s}^{1},\ldots ,k_{s}^{d}\right)$, and each $k_{s}^{i}$ in $\left\{k_{s}^{1},\ldots ,k_{s}^{d}\right\}$ is a *k*-bit random string, each $st_{j}^{\prime }$ in $\tau_{i}$ is indistinguishable from ${\tilde{\tau }}_{i}$ by any PPT adversary. Therefore, $Sim_{1}$ cannot learn extra information from $\tau_{1},\ldots ,\tau_{q}$, which satisfies:

$${\mathrm{Output}}_{A_{1}}^{\mathrm{Real}}\left(\left\{\tau_{1},\ldots ,\tau_{q}\right\}\right)\approx {\mathrm{Output}}_{Sim_{1}}^{\mathrm{Ideal}}\left(\left\{{\tilde{\tau }}_{1},\ldots ,{\tilde{\tau }}_{q}\right\}\right).$$

The sorting network between $A_{1}$ and $A_{2}$ contains ${\left(\log d\right)}^{2}$ levels, and each level contains ${\left(\log d\right)}^{2}$ times of $P_{3}$ protocols. Therefore, the simulation of the sorting network can be reduced to prove $Sim_{1}$ and $Sim_{2}$ can simulate the secure comparison protocol $P_{1}$ with leakage functions. Given the information leaked from $L_{4}$, $Sim_{2}$ can learn the leaked information $\left(\left[\;\left[z_{i}\right]\;\right],\left[\lambda_{i}\right]\right)$ from each round of $P_{3}$ and the rounds number ${\left(\log d\right)}^{2}$. In each round, $Sim_{2}$ can learn $\left(\left[D_{x}\right],\left[D_{y}\right],l\right)$. $Sim_{1}$ and $Sim_{2}$ should simulate $A_{1}$ and $A_{2}$ with $L_{4}$ by all pairs with ${\left(\log d\right)}^{2}$ times in sorting protocol to get the final simulation value. At every pairs *i*, $A_{1}$’s view can be denoted as $view_{A_{1}}=\left(sk_{s},\left[\;\left[z\right]\;\right],∥\lambda ∥\right)$. Given $\left(sk_{s},\left[\;\left[z\right]\;\right],\left[\lambda \right]\right)$, we can build $Sim_{1}$ in the following phases:

- Randomly choose $\tilde{\lambda }$, compute $\left|\right|\tilde{\lambda }\left|\right|$ as $x_{x}\leq x_{y}$;
- Randomly choose $\tilde{z}\leftarrow \left(0,2^{\lambda +l}\right)⋃Z$;
- Encrypt $\tilde{z}$: $\left[\tilde{z}\right]\leftarrow P.Enc_{pk_{s}}\left(z\right)$;
- Output $view_{Sim_{1}}=(sk_{s},l,\left[\tilde{z}\right],||\tilde{\lambda }\left||\right)$.

Since z=x+r, where *x* is a *l*-bits integer and *r* is a l+λ-bits integer, the distribution of $\tilde{z}$ is indistinguishable from *z*. We can get $\left(sk_{s},\left[\tilde{z}\right]\right)\approx \left(sk_{s},\left[z\right]\right)$. Besides, since the distribution of $\tilde{z}$ and *z* are independent of *t*, we can get $(sk_{s},l,\left[\tilde{z}\right]\left|\right|\tilde{\lambda }||)\approx (sk_{s},l,\left[z\right],||\tilde{\lambda }\left||\right)$. In a similar way, at every pairs *i*, $A_{2}$’s view can be denoted as $view_{A_{2}}=\left(({\left[D_{x}\right]}_{i},{\left[D_{y}\right]}_{i},l,pk_{s},r,∥\lambda ∥,\left[z_{l}\right]\right)$. We can build $Sim_{2}$ to simulate $A_{2}$ in the following phases:

- Choose $\tilde{r}\leftarrow \left(0,2^{\lambda +l}\right)⋃Z$;
- Choose two random values $\tilde{\lambda },{\tilde{z}}_{l}$, computes $\left|\right|\tilde{\lambda }\left|\right|,∥\tilde{z}∥$;
- Output $view_{Sim_{2}}=\left(\left[D_{x}\right],\left[D_{y}\right],l,pk_{s},\tilde{r},\left[{\tilde{z}}_{l}\right]\right)$.

In both $view_{A_{2}}$ and $view_{Sim_{2}}$, *r* is extracted from uniform distribution $(0,2^{\lambda +l})⋃Z$, $\left[{\tilde{z}}_{l}\right]$ is the ciphertext of P which is randomness, so $\left(\left[D_{x}\right],\left[D_{y}\right],l,pk_{s}\right)\approx (\left[D_{x}\right],\left[D_{y}\right],l,pk_{s},r,$

$\left[{\tilde{z}}_{l}]\right)$. We can obtain: $view_{A_{2}}$ and $view_{Sim_{2}}$ are computational indistinguishable. What is more, since $\left|\right|\tilde{\lambda }\left|\right|\approx ∥\left[D_{x}\right]\leq \left[D_{y}\right]∥$, $(sk_{s},l,\left[z\right],||\tilde{\lambda }||)\approx (sk_{s},l,\left[z\right],$$∥\left[x_{x}\right]\leq \left[y_{y}\right]∥)$. Due to the semantic security of DGK, $Sim_{1}$ and $Sim_{2}$ can obtain *d* ciphertexts that are unsorted from the leakage function $L_{4}$. Then, $Sim_{1}$ and $Sim_{2}$ can simulate Bathcer’s sorting protocols in ${\left(\log d\right)}^{2}$ times.

Therefore, for all polynomial time $A_{1}$ and $A_{2}$, there exists polynomial time simulators $Sim_{1}$ and $Sim_{2}$ such that:

We can demonstrate that the proposed scheme satisfies adaptive L-semantic security in the random oracle model, which is defined in Definition 2. Theorem 1 proved. □

### 4.2. Revocation Secure

**Theorem** **2.**  *If BE is CPA secure, then the proposed scheme satisfies revocation secure, which is defined in Definition 3.*

**Proof.**  Assuming the advantage of $A_{rev}$ to win ${\mathrm{Exp}}_{A_{rev}}^{\mathrm{Revoke}}\left(1^{k}\right)$ is negligible, we can construct an adversary $A_{be}$, who can break the CPA secure of BE with assist of $A_{rev}$. We will show that if $A_{rev}$ has a non-negligible advantage in ${\mathrm{Exp}}_{A_{rev}}^{\mathrm{Revoke}}\left(1^{k}\right)$, then we can construct an adversary $A_{be}$ that uses $A_{rev}$ as a subroutine to break the CPA secure of BE.

To make the output of ${\mathrm{Exp}}_{A_{rev}}^{\mathrm{Revoke}}\left(1^{k}\right)$ as 1, $A_{rev}$ needs to provide a valid search token. To achieve that, $A_{rev}$ must know $st_{i}$. A new value of $st_{i}$ is randomly selected and encrypted by $\mathrm{BE}.Enc_{bpk_{i}}\left(st_{i},F_{i}\setminus u_{j}\right)$ at each time a user is revoked from the system, where $F_{i}\setminus u_{j}$ is the new friends index. $A_{rev}$ then broadcast this encrypted value to all users. BE’s security ensures that only a non-revoked friend of $U_{i}$ can decrypt this ciphertext to obtain $st_{i}$ with overwhelming probability. Hence, the adversary can only create a valid search token if he is a valid friend of $U_{i}$, or he will break the security of BE. That is, the probability that a random bit string is valid is $2^{-k}$. It means that the adversary will not be able to produce a valid token with non-negligible probability.

Let C be the challenger for the adversary $A_{be}$ against BE, $A_{be}$ will act as the challenger for $A_{rev}$:

1. C runs $\mathrm{BE}.KeyGen(1^{k})$ to generate keys $\left(msk_{be},bpk_{i}\right)$. $A_{be}$ initializes $F_{i}$, randomly chooses a *k*-bit string $st_{i}$, and sends $\left(st_{i},F_{i}\right)$ to C. C runs $\mathrm{BE}.E{\mathrm{nc}}_{bpk_{i}}\left(st_{i},F_{i}\right)$ to generate $st_{S_{1}}$, and sends it to $A_{be}$. $A_{be}$ runs KeyGen to generate $K_{i}$, runs Join to generate $k_{S_{1}}^{i}$, where $K_{i}$ does not include $k_{be}$.
2. $A_{be}$ issues a query to C for the secret key of $A_{rev}$. C runs $\mathrm{BE}.J{\mathrm{oin}}_{msk_{i}}\left(A_{rev}\right)$ to generate $k_{A_{rev}}$, sends $k_{A_{rev}}$ to $A_{be}$. To fully enroll $A_{rev}$ as a valid friend, the state ciphertext also needs to be updated by $A_{be}$. $A_{be}$ send $F_{i}$ and a newly generated $st_{i}$ to C, C runs $\mathrm{BE}.Enc_{bpk_{i}}\left(st_{i},F_{i}\right)$ to generate new $cst_{i}$. $A_{be}$ runs Grant to generate the key $k_{A_{rev}}^{i}$ of $A_{rev}$.
3. $A_{be}$ runs **Initial** to generate graph G, and sends $k_{A_{rev}}^{i}$ and G to $A_{rev}$. $A_{rev}$ can access to oracles $O_{\mathrm{Grant}}$ and $O_{\mathrm{Revoke}}$.
4. $A_{be}$ revokes $A_{rev}$ by running Revoke, $A_{be}$ runs **Revoke** a second time in order to produce two values $st_{i0}\leftarrow {\left\{0,1\right\}}^{k}$ and $st_{i1}\leftarrow {\left\{0,1\right\}}^{k}$ for $st_{i}$, and sends $st_{i0}$ and $st_{i1}$ to C as the challenge value for $A_{be}$, along with a set of no revoked friends $F_{i}$ of $A_{rev}$.
5. C selects a bit b∈{0,1}, uses $\mathrm{BE}.Enc_{bpk_{i}}\left(st_{ib},F_{i}\right)$ to encrypt $st_{ib}$ and generates ${cst_{i}}_{b}$, sends ${cst_{i}}_{b}$ to $A_{be}$ as the challenge ciphertext for the CPA secure of BE. $A_{be}$ sends ${cst_{i}}_{b}$ to $A_{rev}$ as the challenge ciphertext of ${\mathrm{Exp}}_{A_{rev}}^{\mathrm{Revoke}}\left(1^{k}\right)$.
6. $A_{rev}$ generates token τ, and sends τ to $A_{be}$. Since the advantage for $A_{rev}$ to win ${\mathrm{Exp}}_{A_{rev}}^{\mathrm{Revoke}}\left(1^{k}\right)$ is non-negligible, the probability of validity of τ is non-negligible.
7. If $t_{0}\neq ⊥$, then **Search** stops. According to the following situations, $A_{be}$ outputs its guess for *b*:
  - If $t_{0}\neq ⊥$, this tells $A_{be}$ that $st_{i0}$ was used to generate the token, $A_{be}$ outputs its guess for *b* as $b^{\prime }=0$;
  - Of $t_{1}\neq ⊥$, this tells $A_{be}$ that $st_{i1}$ was used to generate the token, $A_{be}$ outputs its guess for *b* as $b^{\prime }=1$.

From the above analysis, the advantage of $A_{be}$ to break the CPA secure of BE can be computed as ${\mathrm{Adv}}_{A_{be}}^{\mathrm{BE}}\left(1^{k}\right)$:

$$\begin{array}{cc} {\mathrm{Adv}}_{A_{be}}^{\mathrm{BE}}\left(1^{k}\right) & =|[\left(\mathrm{Pr}\left[\left(t_{0}\vee t_{1}\right)\neq ⊥\right]\;\cdot \;1-\frac{1}{2}\right)+\left(\mathrm{Pr}\left[\left(\left(t_{0}\wedge t_{1}\right)\neq ⊥\right]\;\cdot \;\frac{1}{2}-\frac{1}{2}\right)\right|\\ \; & =|\delta \;\cdot \;1+\left(1-\delta \right)\;\cdot \;\frac{1}{2}-\frac{1}{2}|\\ \; & =|\frac{\left(\delta +1\right)}{2}-\frac{1}{2}|\\ \; & =\frac{\delta }{2}. \end{array}$$

Since the advantage δ of $A_{rev}$ to win ${\mathrm{Exp}}_{A_{rev}}^{\mathrm{Revoke}}\left(1^{k}\right)$ is non-negligible, the advantage $\frac{\delta }{2}$ of $A_{be}$ to break the CPA security of BE is non-negligible, which contradicts the CPA security of BE. Therefore, there exists no $A_{rev}$, who can win ${\mathrm{Exp}}_{A_{rev}}^{\mathrm{Revoke}}\left(1^{k}\right)$ with non-negligible probability, and the proposed scheme satisfies revocation security as defined in Definition 3. Theorem 2 proved. □

## 5. Theoretical Analysis

The complexity analysis is shown in Table 1, where *n* is the maximum amount of enrolled users and *d* is the maximum amount of each user’s friends. We compare our scheme with the related privacy-preserving location-based query schemes [15,18,20] in Table 2. Due to the significant differences among the existing schemes in application scenarios, secure models, evaluation indicators and other factors, we focus on comparing characteristics and security.

For result accuracy, [15] achieves differential privacy for location information using linear programming techniques. It is specifically designed for simple computation that cannot provide accurate encrypted distance sorting. Ref. [20] uses a dynamic location grid structure to cluster users close to each other. However, the search results in [15,20] have a specific rate of false positives, which are suitable for similarity search. Our scheme and [18] use Euclidean distance to calculate the encrypted distance to achieve precise secure sorting. Ref. [18] focuses on searching the number of points of interest in a specific location area; our scheme sorts the distances based on the proven-secure comparison protocol. In terms of security, Ref. [18] protects location search privacy by way of private information retrieval (PIR). Although it adopts the anchor technology to improve search efficiency, it still has a certain communication overhead. Ref. [20] achieves sort privacy by assuming the server only performs the search, and the user performs the result sorting. As a result, the above methods each sort privacy but lead to high computation or communication costs.

Besides, compared with other schemes, our scheme also has a flexible access control mechanism. Moreover, our scheme achieves a constant-time computation cost and communication cost when updating friends and encrypting locations, and a user only needs to store key-related information locally. Therefore, we can demonstrate that our proposed scheme has both a very light user workload and a moderate server workload while being secure against the honest-but-curious adversary. In nowadays’s mobile social networking environment, the user-side lightweight device’s storage and computation cost should be minimized as much as possible. As a consequence, the proposed scheme is more suitable for the real-life thin clients MSNs deployment scenario.

## 6. Implementation

We implement and analyze the performance of our scheme. The experiments were run on several computers with Linux Ubuntu 18.04.2 64-Bit Version with Inter(R) Core(TM) I7-2600 quad-core processor (3.4 ghz) and 8 GB memory, which were installed on VMware Workstation in the LAN in C++ language. One of the computers acted as the server-end and the others acted as user-ends, respectively. We implemented a job allocation mechanism in the server-end that the computer acted as the master server and used threads to simulate the collaborated server that performed the assistant job. Each user-end stored the user’s keys locally and interacted with the server-end. To submit a search request, a user-end only communicated with the master server.

In the simulation experiments, the security parameter *k* was set to 256 bits. We chose SHA256 in the OpenSSL library [32] for the pseudo-randomness function, and used the Relic library [33] to implement Paillier and GM homomorphic encryption. To implement the scheme more securely, we improved the modulus *n* of the Paillier and GM to 1024 bits. Besides, we used BGW2 [26] to implement public-key broadcast encryption. The key length in the above public encryption methods was set to be 1024 bits.

We conducted data simulations based on real-world data sets, which came from the newest version of the Enron email dataset [34], where we randomly selected 1000 accounts as the total users set. We represented users’ friendships in the form of linked contacts. We selected a random integer in (10,50) to simulate the user’s location’ value, which was updated periodically. Moreover, we initialized the social network graph structure G with 1000 vertexes and 3831 edges that contained the above data and used a unique value to identify each vertex (user) in $Z_{k}$. We did not record the network communication time during all the experiments since it depends on the user-end and the server-end’s network connection. Each data point in the experiments was obtained after being repeated 50 times to generate the average value.

### 6.1. Storage Analysis

We first analyzed the storage overhead of our scheme. Table 3 shows the comparison between the encrypted G and unencrypted G of the generation time and the server’s storage cost in the trend of the number of users increases. It can be seen that the server’s storage cost increased almost linearly with the increase of the number of users. Since we used symmetric encryption to encrypt location, compared with the Paillier homomorphism ciphertext, the inflation rate of the symmetric ciphertext of the location decreased significantly, which is consistent with the theoretical analysis. Therefore, the proposed scheme achieves the trade-off of users’ location confidentiality and search privacy with the acceptable additional storage cost.

### 6.2. Communication

In terms of communication, we mainly analyzed the amount of data transformed between (1) $U_{i}$ and $S_{1}$ and (2) $S_{1}$ and $S_{2}$ in **Search** protocol. Theoretically, when $U_{i}$ requests to search *k*-nearest neighbors among his *d* friends, $U_{i}$’s communication overhead increases almost linearly with *k*. When $S_{1}$ and $S_{2}$ interact with each other to compute the distance from the total of *d* friends’ location ciphertexts, the data size of the communication between them is $O({\left(\log d\right)}^{2})$.

**Figure 2:**
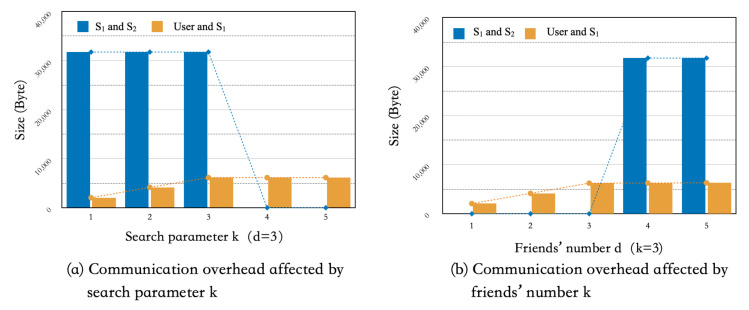
Communication overhead.

Figure 2a,b shows the relationship between the two types of communication overhead in the experiment with the increasing trends of the friends’ number *d* and the search parameter *k*, respectively. In general, the amount of data transmission required by the user in **Search** protocol is positively related to *k*. When *k* increases to a particular value (greater than *d*), the data transmission volume tends to be stable. The communication overhead between $S_{1}$ and $S_{2}$ is mainly positively related to *d*, but independent of the increase of *k*. Moreover, the distance computation sub-procedure requires several rounds of interactions, so the amount of communication overhead between servers is relatively large, which is consistent with the theoretical analysis.

### 6.3. Search Time

**Figure 3:**
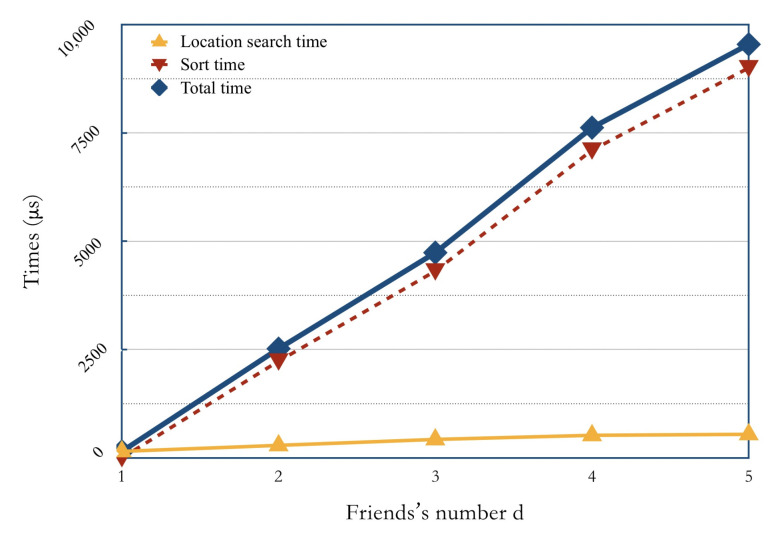
Search time overhead.

We also analyzed the primary source of the search time overhead for Search protocol. First, we divided the Search protocol at the server-end into two sub-procedures of location search and distance sort. Figure 3 shows the relationship between search time and the number of friends *d*. In Figure 3, the total time overhead of Search protocol is shown in the blue curve, the time overhead to extract and re-encrypt location ciphertext is shown in the yellow curve, and the time overhead to compute and sort the encrypted distance is shown in the red curve.

From Figure 3, we can see that the time overhead of the two sub-procedures in the Search protocol generally increases with the increasing trend of *d*. Specifically, the location search time is far lower than the distance sort time, and with *d* increases to 4, the curve growth is slowing down. The distance sort time has a stable approximate linear relation with *d*. Therefore, it can be concluded that the computation and comparison of encrypted distances are two primary time-overhead sources of the Search protocol, which is consistent with the theoretical analysis.

### 6.4. Scalability

**Figure 4:**
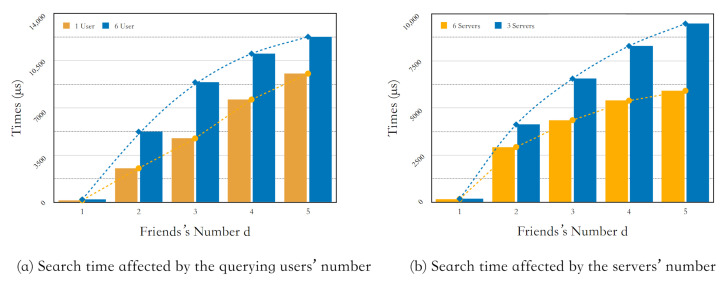
Scalability.

In terms of scalability, we first analyzed the impact of the search users’ number who submit search requests in parallel on the time overhead of the Search protocol. To be specific, we deploy one host to simulate one user to execute the Search protocol and record the total time overhead. Then we deploy six hosts to simulate six users to repeat the same experiment and compare the results. It is worth mentioning that, when recording the time of multi-user search, multiple user-ends simultaneously send the search requests to the server-end. We record the start and end time when the server-end receives the search request until it completes each user’s search. Figure 4 shows the relationship between the parallel search users’ number and Search protocol’s total time. It can be seen that one user’s search time is slightly lower than six parallel users’ search times. The former is approximately in a stable linear relation with *d*, and the latter slows down to a constant level with the increase of *d*. From the trend it can be concluded that, with the number of search users *d* increasing, its impact on search time overhead is weakened, and it further weakens the influence of the increasing number of friends on the search time. Therefore, the multi-user parallelism has a weak impact on search time overhead, which helps the scheme to achieve a certain level of scalability.

Besides, we analyzed the influence of the expandable number of remote servers on the search time overhead. First, we deployed three servers to execute the Search protocol for six users simultaneously and recorded the total time overhead. Then we deployed six servers to repeat the same experiment and compare the results. Figure 4 shows the relationship between the number of servers and the search time. It can be seen that the search time of 6-server deployment is significantly lower than the running time with 3-server deployment, and the former’s growth was slowed down to a constant level after *d* reaches 4, but the latter’s growth takes an approximately linear relationship with the number of friends steadily. Therefore, it can be concluded that deploying multiple servers to perform parallel searches can reduce the search time overhead and further weaken the influence of the increasing number of friends on the search time.

**Remark** **1.**  *It is worth pointing out that the search process’s main computation cost is the homomorphic encryption/decryption operation and broadcast decryption operation. The computation efficiency is closely related to the selected parameters of the underlying algorithms. The server-end implementation can also be optimized to reduce the search time by using multiple threads for distance sorting and using approximate sorting algorithms, and so forth. In our experiment, we did not adopt any optimization method. The server was allowed to complete all the computation steps in a single thread in each phase to reflect the scheme’s original execution efficiency faithfully.*

## 7. Conclusions

Aiming at the problem of location privacy disclosure in MSNs, we propose a privacy-enhancing *k*-nearest neighbors search scheme over MSNs. We deploy a dual-server collaborative architecture and design an encrypted location-oriented *k*-neighbor search protocol based on secure multi-party computation and homomorphic encryption. Our scheme achieves accurate nearby friends retrieval while protecting the geo-location and the distance order from revealing them to the servers. We propose a lightweight dynamic friends management mechanism based on public-key broadcast encryption to satisfy the fine-grained access control requirement. It enables users to grant/revoke a friend’s location search right without updating others’ keys and achieves constant-time identity authentication. The scheme satisfies adaptive L-semantic security and revocation security under the random oracle model. Compared with the works on single server architecture, the proposed scheme reduces the communication cost between users and the server and prevents location information leakage, which achieves a trade-off of the location availability and privacy.

## Figures and Tables

**Table 1:** Complexity analysis.

	${\mathrm{Stor}}_{u}$	${\mathrm{Stor}}_{S_{1}}$	${\mathrm{Stor}}_{S_{2}}$	${\mathrm{Comp}}_{u}$	${\mathrm{Comp}}_{S_{1}}$	${\mathrm{Comp}}_{S_{2}}$	${\mathrm{Comm}}_{u}$	${\mathrm{Comm}}_{S_{1}}$	${\mathrm{Comm}}_{S_{2}}$
**Register**	O(1)	O(*n*)	O(*n*)	O(1)	–	–	O(1)	O(1)	O(1)
**Grant**	O(*d*)	O(nd)	–	O(*d*)	–	–	O(*d*)	O(nd)	–
**Revoke**	–	–	–	O(1)	O(1)	–	O(1)	O(1)	–
**LocUpdate**	–	O(*n*)	–	O(1)	–	–	O(1)	O(*n*)	–
**Search**	–	–	–	O(*d*)	O(d+(log*d*)${}^{2}$)	O(d+(log*d*)${}^{2}$)	O(*k*)	$O({\left(\log d\right)}^{2})$	$O({\left(\log d\right)}^{2})$

Stor: storage complexity; Comp: computation complexity; Comm: communication complexity.

**Table 2:** Properties comparison.

	Accuracy	Evaluation Method	Dynamic	Cryptography tool	SP	LP	AC	Rank Model
[16]	✓	Euclidean distance/Anchor points	×	PIR/P	✓	✓	×	–
[17]	×	Linear Programming	✓	HMAC	✓	✓	×	–
[21]	×	Dynamic Grid	×	HMAC	✓	×	×	User
Ours	✓	Squared Euclidean distance	✓	P/GM/BE	✓	✓	✓	2 servers

SP: Search Privacy; LP: Location Privacy; AC: Access Control.

**Table 3:** Storage cost.

	Unencrypted G		Encrypted G		
**Vertex**	**Storage (kb)**	**GenTime (s)**	**Storage (kb)**	**GenTime (s)**	**Inflation Rate**
200	18.752	0.423	57.506	2.359	306.665%
400	38.101	0.477	115.302	2.941	302.622%
600	57.460	0.514	174.379	3.316	303.478%
800	74.677	0.538	225.039	3.770	301.349%
1000	95.988	0.575	289.864	4.113	301.979%
